# Deformation twinning mechanism in hexagonal-close-packed crystals

**DOI:** 10.1038/s41598-018-37067-8

**Published:** 2019-01-24

**Authors:** Shan Jiang, Zhongtao Jiang, Qiaowang Chen

**Affiliations:** Research Institute for New Materials and Technology, Chongqing University of Arts and Sciences, Chongqing, 402160 P. R. China

## Abstract

The atomic structure of {10 $\bar{{\bf{1}}}$ 2} twin boundary (TB) from a deformed Mg-3Al-1Zn (AZ31) magnesium alloy was examined by using high-resolution transmission electron microscopy (HRTEM). By comparing the lattice structure of TB with the previously established model, a kind of special atomic combinations, here named primitive cells (PCs), were discovered at the TB. The PCs reorientation induced mechanism of twinning in hexagonal-close-packed (HCP) crystals was hence verificated. Meanwhile, the relationship between the misorientation of adjacent layers of PCs and the width of TB was discussed. The verification of the mechanism clarifies the twinning mechanism in HCP crystals and opens up opportunities for further researches.

## Introduction

The deformation twinning from HCP crystals has been heavily researched^1–6^ due to the role it plays in the dominant deformation mode and the strengthening mechanism of materials^7–10^. The details of a number of phenomena relevant to twinning, including the micro-structure of TBs, the TB migration characteristics, and the twin nucleation remain obscure due to the lack understanding of the twinning mechanism on an atomic scale. The researches on HCP twinning can be divided into two levels of closely related topics: the structure of TBs^11,12^ and the law of atomic migration in twinning. The TBs were recently considered to be composed of a mixture of dislocations^13–15^ and the dynamic twinning process was considered to be governed by either the glide of defects on their twin planes^16–19^, or shuffling^20–23^. The authors of this paper had established a new theoretical model based on “atomic groups rotation” to describe the atomic motion in HCP twinning elsewhere^24–26^, which was specially concerned with the integrity of atomic motion. In this paper, the theoretical hypothesis is to be verificated and discussed. To avoid ambiguity the expression “atomic groups” was replaced by “PCs”.

## Results

**Figure 1:**
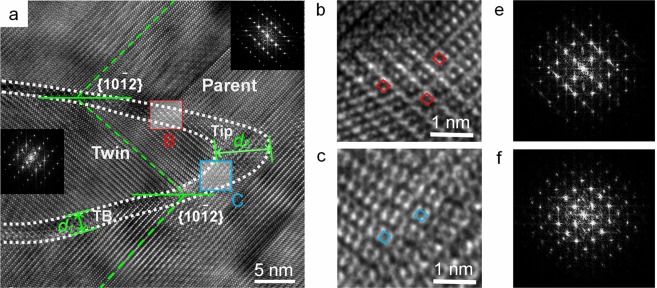
HRTEM image of the deformed AZ31 alloy containing a {10 $\overline{1}$ 2} twin (**a**) and enlarged views of the areas enclosed by the box B (**b**) and box C (**c)**. (**e**,**f**) are FFT patterns of (**b**,**c**), respectively.

The picture recording of the atomic array around the TB of a {10$\overline{1}$2} twin from the deformed AZ31 alloy was obtained by HRTEM detection (Fig. 1a). The image was divided into three parts consisting of the parent, twin, and TB. The orientations of the parent and the twin were symmetrical about the {10$\overline{1}$2} twinning plane, but not the TB. The TB was not an imagined thin interface composed of single-layered atoms, but a large range of distorted lattice regions composed of muti-layered atoms. Around the TB, some atoms gathered together closely, making them distinguishable in the formation of some specific atomic combinations, as denoted by the diamonds in Fig. 1b,c. According to the geometric shape characteristics, these atomic combinations are identified as the proposed “PCs”. To clarify that the PCs observed in the TB region in Fig. 1 are not artifacts, the corresponding Fast-Fourier-Transform (FFT) patterns of Fig. 1b,c (Fig. 1d,f) together with the FFT patterns of the neighboring parent and twin regions (Fig. 1a) were provided. The result indicates that the FFT patterns of the PCs are not simply a combination of the FFT patterns of the twin and parent regions.

**Figure 2:**
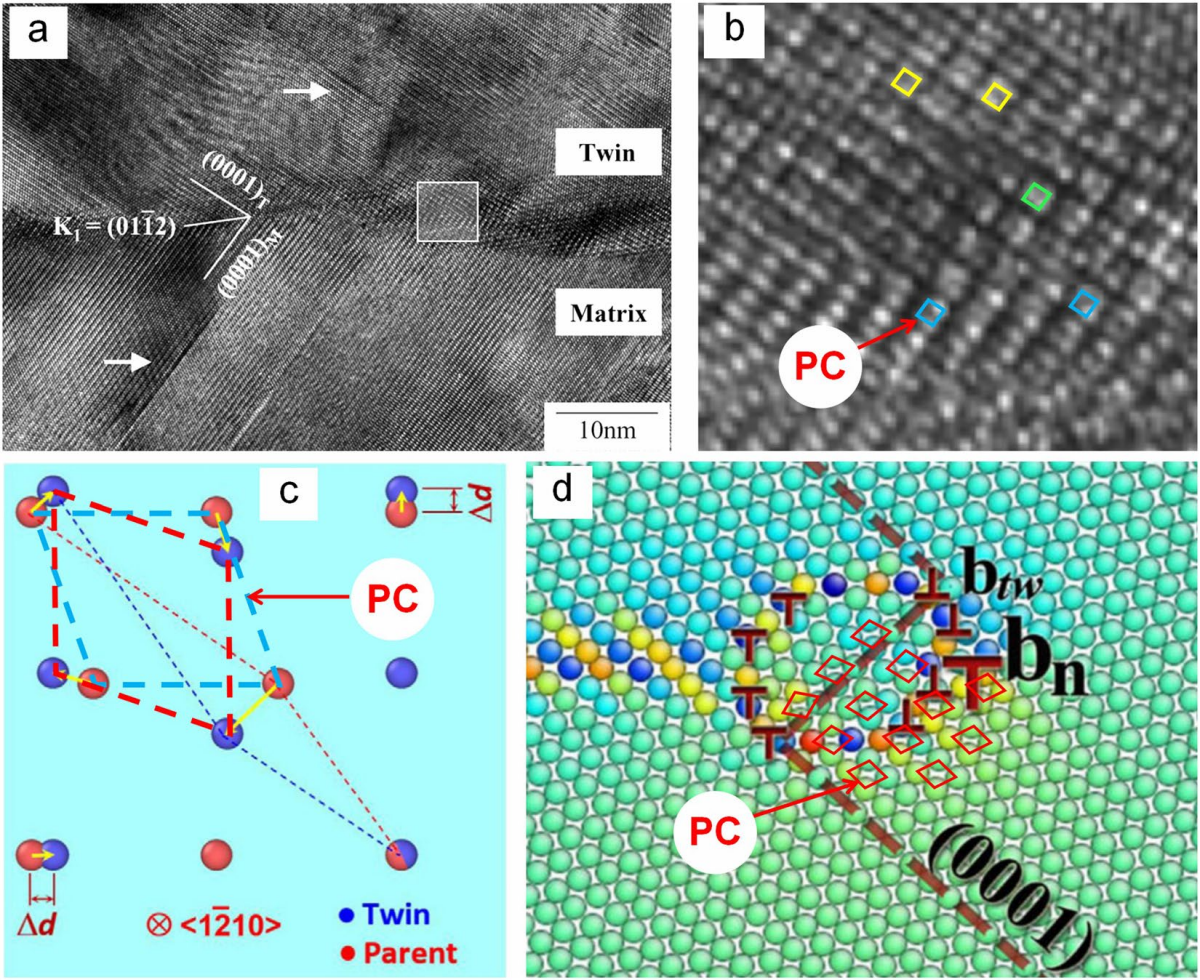
Similar discoveries in other literature: (**a**) {10 $\overline{1}$ 2} TB of Mg_97_Zn_1_Y_2_ magnesium alloy detected by HRTEM; (**b**) enlargement of the block area in (**a**); (**c**) shuffles in projection view along the 〈1 $\overline{2}$ 10〉 zone axis in HCP structures; and (**d**) a partial dislocation *b*_*n*_ in the {10 $\overline{1}$ 2} twin nucleation of Mg obtained by atomistic simulations.

Some related literature can also verify the existence the PCs in HCP twinning. Figure 2a was the HRTEM image of {10$\overline{1}$2} TB of the Mg_97_Zn_1_Y_2_ alloy^27^. Figure 2b is the enlarged view of the area enclosed by the box in Fig. 2a, where the reorientating PCs can be clearly observed, as indicated by the diamonds. Figure 2c was a schematic of HCP {10$\overline{1}$2} twinning by shuffle mechanism^28^, where the PC can also be distinguished as indicated by the added dotted line parallelograms. Figure 2d was the diagram of {10$\overline{1}$2} twinning nucleation in Mg obtained by atomistic simulations^29^. Around the TB, the PCs can also be marked off, as indicated by the diamonds. In summary, the appearance of PCs in HCP twinning is universal.

## Discussion

According to the PC model, the PCs were considered to rotate as a whole to induce the migration of TB. However, the specific mechanism depends on the structure of the TB. As mentioned above, the TB was composed of layers of PCs oriented between the parent and the twin. Although the total misorientation of PCs between the parent and the twin is fixed, the misorientation between adjacent layers of PCs depends on the width of TB, namely the number of layers of PCs.

**Figure 3:**
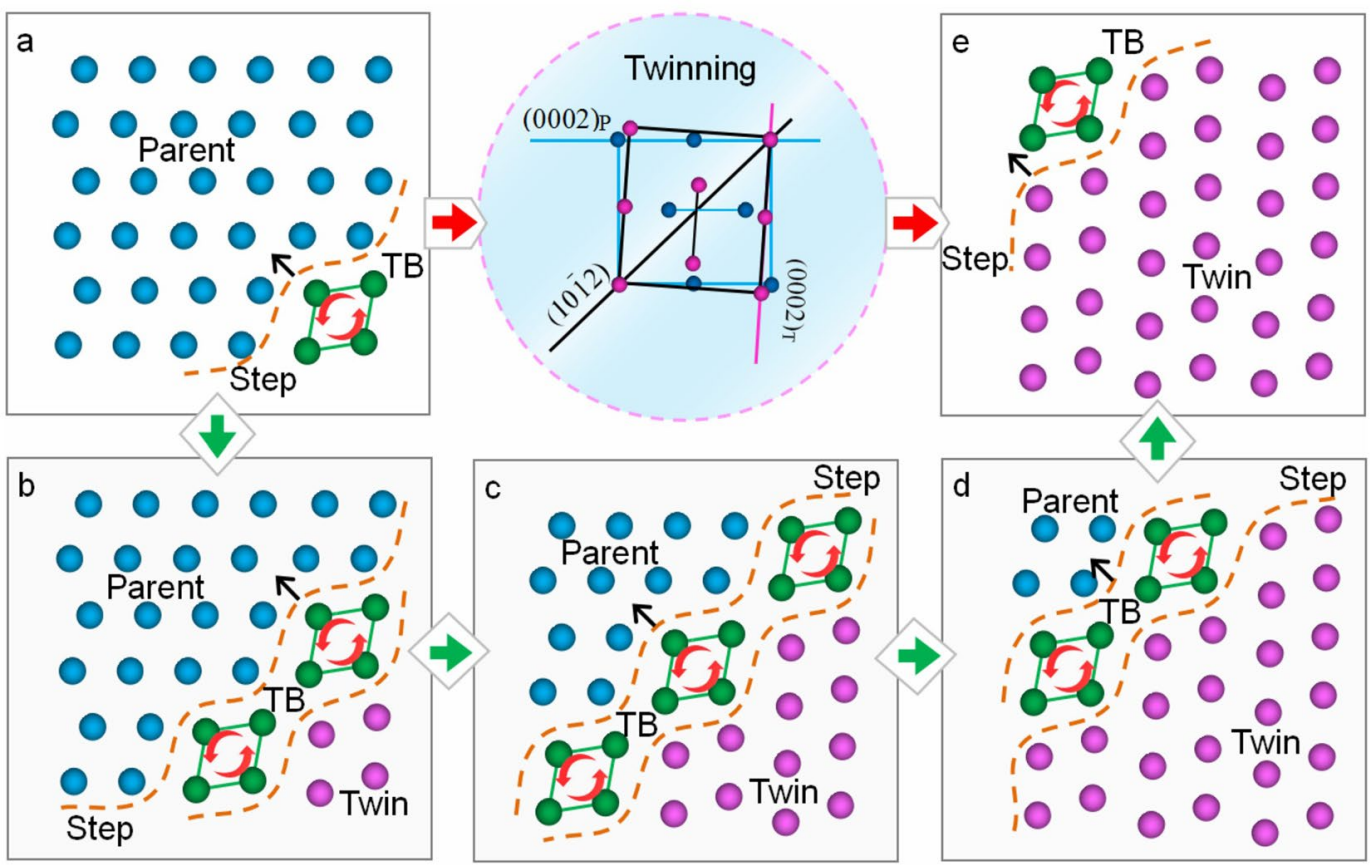
TB migration illustration of HCP {10 $\overline{1}$ 2} twining where the TB sweeps across five layers of PCs. From (**a**) to (**e**) the first to fifth layer of PCs serve as the TB, respectively.

The simplest TB structure is that there is only one layer of PCs serving as the TB, as indicated in Fig. 3. The orientation of PCs at the TB is between that at the parent and the twin. Figure 3(a–e) illustrates the process of the migration of the TB, where the reorientation of PCs make the TB sweep over the lattice and transform the parent into the twin. The border between adjacent layers of PCs presents ‘steps’ shape due to the lattice distortion (Fig. 3b–f), which was named twinning dislocation elsewhere^4^. When the PCs in Fig. 3 rotate anticlockwise the TB migrates along the arrows, namely, twinning occurs. As a requirement for the activation of PC rotation, the PCs must break through the lattice resistance sustained from the surrounding atoms. The resistance was closely linked to the misorientation *θ* between two adjacent layers of PCs (suppose that the misorientation was uniform). For the TB composed of a single layer of PCs, *θ* = *α*/2, *α* denotes the total misorientation of PCs from parent to twin. When this equation is applied to {10$\overline{1}$2} twinning in magnesium, *α* ≈ 15.9°, *θ* ≈ 8°.

**Figure 4:**
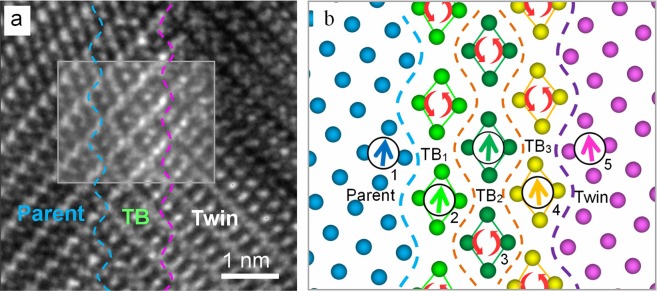
(**a**) HRTEM image of AZ31 alloy containing a number of layers of gradually reorientating PCs. (**b**) Schematic of distribution of orientations of PCs in the area enclosed by the box in (**a**).

Often cases vary from the above hypothesis. Because TB is usually composed of muti-layered PCs. Figure 4a presents a HRTEM image of {10$\overline{1}$2} TB containing three layers of PCs in AZ31 alloy. Figure 4b is the schematic of distribution of PCs orientations corresponding to the box in Fig. 4a. The arrows denote the orientations of the PCs. As the arrows show, the PCs exhibit a strong gradual reorientating inclination from the parent to the twin. The five arrows form four misorientations, *α*_1_, *α*_2_, *α*_3_, and *α*_*4*_, between every two adjacent layers (Fig. 4c). Thus, the total misorientation of PCs from parent to twin can be expressed as:

1 $$\alpha ={\alpha }_{1}+{\alpha }_{2}+{\alpha }_{3}+{\alpha }_{4}$$

If these reorientating PCs were well-distributed from parent to twin, the above equation changes to:

2 $$\alpha =4\theta ,\,or\,\theta =\alpha /4$$

where *θ* is the average misorientation between adjacent PCs. By extension, the value of *θ* for a TB contains *n* layers of PCs can be expressed as:

3 $$\theta =\alpha /(n+1)$$

Thus, when this equation is applied to the case seen in Fig. 4b (where *α* ≈ 15.9°, n = 3), *θ* ≈ 4°; and when it is applied to the TB with the width of *d*_2_ that consisted of approx. 15 layers of PCs, *θ* ≈ 1°. (Fig. 1a). Apparently, the resistance for twinning to break through decreases with the increasing number of layers of PCs. In another word, an increased number of layers of PCs can reduce the critical resolved shear stresses (CRSS) required in twinning activation. These conclusions may help to explain or predict phenomena regarding the TB movement. For example, for the TB shown in Fig. 1a the width of TB at the tip was larger than at the edge (Fig. 1a), which resulted in a more rapid growth along the longitudinal direction. The establishment of PC model is also helpful to explain the mechanism of twin nucleation. Since a twin was formed from a nucleus during TB migration from inside to outside, an inverse process can restore the original appearance of the nucleus. The verification of the PC mechanism opens the opportunity for further researches relevant to twinning.

## Conclusions

The atomic combinations discovered at the TB were identified as proposed PCs in accordance to their characteristics, verifying the PC induced mechanism. The twinning process was induced by the rotation of the PCs. To accomplish this, the PCs must overcome the resistance from the surrounding lattice that was closely related to the CRSS. This was not determined by their total rotational angle from the parent to the twin, but rather from the misorientation between adjacent PCs.

## Methods

A cuboid sample was cut from a hot-rolled AZ31 sheet with a dimension of 30 × 30 × 22 mm^3^ in the rolling direction, transverse direction, and normal direction. The sample was compressed by about 7% at a strain rate of ~10^−3^ s^−1^ at room temperature, with the loading direction parallel to the rolling direction. The HRTEM sample was prepared via low temperature ion thinning. A FEI Tecnai F30-G2 electron microscope with a voltage of 300 kV was used to carry out the HRTEM observations.
